# Antiviral Effects of Clinically-Relevant Interferon-α and Ribavirin Regimens against Dengue Virus in the Hollow Fiber Infection Model (HFIM)

**DOI:** 10.3390/v10060317

**Published:** 2018-06-09

**Authors:** Camilly P. Pires de Mello, George L. Drusano, Jaime L. Rodriquez, Ajeet Kaushik, Ashley N. Brown

**Affiliations:** 1 Institute for Therapeutic Innovation, Department of Medicine, College of Medicine, University of Florida, Orlando, FL 32827, USA; Camilly.Ribeiro@medicine.ufl.edu (C.P.P.d.M.); George.Drusano@medicine.ufl.edu (G.L.D.); Jaime.Rodriquez@medicine.ufl.edu (J.L.R.); 2 Center for Personalized Nanomedicine, Institute of Neuroimmune Pharmacology, Department of Immunology, Herbert Wertheim College of Medicine, Florida International University, Miami, FL 33199, USA; akaushik@fiu.edu

**Keywords:** Dengue virus, antiviral therapy, combination therapy, ribavirin, interferon-α, hollow fiber infection model, mathematical modeling

## Abstract

Dengue virus (DENV) is the most prevalent mosquito-borne viral illness in humans. Currently, there are no therapeutic agents available to prevent or treat DENV infections. Our objective was to fill this unmet medical need by evaluating the antiviral activity of interferon-α (IFN) and ribavirin (RBV) as a combination therapy against DENV. DENV-infected Vero and Huh-7 cells were exposed to RBV and/or IFN, and the viral burden was quantified over time by plaque assay. Drug-drug interactions for antiviral effect were determined by fitting a mathematical model to the data. We then assessed clinically-relevant exposures of IFN plus RBV using the hollow fiber infection model (HFIM) system. RBV monotherapy was only effective against DENV at toxic concentrations in Vero and Huh-7 cells. IFN, as a single agent, did inhibit DENV replication at physiological concentrations and viral suppression was substantial in Huh-7 cells (Half maximal effective concentration (*EC*_50_) = 58.34 IU/mL). As a combination therapy, RBV plus IFN was additive for viral suppression in both cell lines; however, enhancement of antiviral activity at clinically-achievable concentrations was observed only in Huh-7 cells. Finally, clinical exposures of RBV plus IFN suppressed DENV replication by 99% even when treatment was initiated 24 h post-infection in the HFIM. Further evaluation revealed that the antiviral effectiveness of the combination regimen against DENV is mostly attributed to activity associated with IFN. These findings suggest that IFN is a potential therapeutic strategy for the treatment of DENV.

## 1. Introduction

According to the World Health Organization it is estimated that over 300 million dengue virus (DENV) infections occur every year and 3.9 billion people in 128 endemic countries are at risk for infection, especially in the Americas, Southeast Asia, and Western Pacific regions [1,2]. This mosquito-borne virus is a positive-sense RNA virus that belongs to *Flaviridae* family [3]. There are four prevalent serotypes of DENV (DENV1, DENV2, DENV3, and DENV4) and, recently, a rare fifth variant was isolated from a human clinical sample [4,5]. The four prevalent serotypes of DENV can cause nearly identical clinical manifestations, with primary infection usually resulting in dengue fever; a self-limiting febrile illness characterized by severe muscle and joint pain. Secondary infection with a different serotype can exacerbate disease due to antibody-depend enhancement (ADE), leading to more serious clinical consequences, including dengue hemorrhagic fever (DHF) and dengue shock syndrome (DSS) [6,7]. Patients with DHF experience vascular permeability resulting in plasma leakage. In DSS, the plasma leakage is so severe that shock can occur, which increases the risk of multi-organ failure and death [8,9].

There is currently no antiviral therapy available for DENV. Treatment protocols are only supportive and directed at relieving the symptoms of DENV disease [10]. Dengvaxia^®^, a vaccine developed by Sanofi Pasteur, was approved for the prevention of DENV in 10 countries [11]; however, the efficacy of this vaccine and the ramifications of vaccination on the enhancement of disease caused by other co-circulating flaviviruses is still unknown. Recent reports have shown that vaccine effectiveness is dependent on previous infection with DENV. Individuals are protected from recurrent DENV infection if they had been infected prior to vaccination, but those who acquire a primary infection after vaccination have an increased risk of severe disease [12].

In light of the limitations of vaccination, antiviral therapy still plays an important role in treating DENV-infected patients. Statins, chloroquine, iminosugars, and corticosteroids have demonstrated promising anti-DENV activity both in vitro and in vivo, but ultimately failed in clinical trials [13,14,15,16]. The reason for the failure of these antiviral drugs is not clear, but highlights the complex nature of treating DENV infections.

Ribavirin (RBV) and interferon-α (IFN) are two broad-spectrum antiviral agents that are approved by the Food and Drug Administration (FDA) for the treatment of Hepatitis C virus (HCV), a virus that belongs to the same family as DENV. RBV is a nucleoside analog that functions through many different mechanisms including inhibiting the viral RNA-dependent RNA-polymerase. IFN is a cytokine produced by cells that activate a signaling pathway for antiviral response, making neighboring cells refractory to viral infection [17]. Others have demonstrated that RBV and IFN have antiviral activity as single agents against DENV and other related flaviviruses [18,19,20,21]. Moreover, it has been shown that RBV has the ability to enhance IFN activity when administered in combination for the treatment of HCV [22].

Here, our objective was to assess the antiviral activity of RBV and IFN as a monotherapy and in combination against DENV2. Since RBV and IFN are already FDA-approved for human use, extensive information on toxicity, pharmacokinetic profiles, and pharmacology are available for both agents. We incorporated this information into our analyses to determine the therapeutic potential of clinically-relevant concentrations of RBV and/or IFN as a treatment strategy for DENV infections.

## 2. Materials and Methods

### 2.1. Cells, Viruses, and Compounds

Vero cells (African green monkey kidney cells) were cultured in Eagle’s minimum essential medium (MEM Corning Cellgro, Tewksbury, MA, USA) and Huh-7 cells (human hepatocarcinoma cells) were maintained in Dulbecco’s Modified Eagle’s Medium high glucose (DMEM; Hyclone, Logan City, UT, USA), each supplemented with 5% fetal bovine serum (FBS; Sigma Aldrich, St. Louis, MO, USA) and 1% penicillin-streptomycin solution (Hyclone, Logan City, UT, USA). Cells were maintained at 37 °C, 5% CO_2_ and split two times a week to maintain subconfluency.

Dengue virus serotype 2 (DENV2), strain New Guinea C was propagated on Vero cells for six days. The collected supernatants were clarified by high speed centrifugation, aliquoted in tissue culture medium supplemented with 20% FBS and stored at −80 °C. Stock viral titer was quantified by plaque assay on Vero cells. DENV2 was chosen for these experiments because this serotype replicates the most efficiently in tissue culture compared to serotypes 1, 3, and 4, as robust viral replication kinetics are optimal for antiviral evaluations.

IFN was obtained from PBL assay science (Piscataway, NJ, USA) and RBV was purchased from Tokyo Chemical Industry Co. Ltd. (Portland, OR, USA). Compounds were stored according to the manufacturer’s recommendation. For each assay, drug stocks of 10 g/L for RBV in sterile deionized water and 2 × 10^5^ IU/mL for IFN diluted in PBS + 0.1% bovine serum albumin (BSA) were freshly prepared.

### 2.2. Cytotoxicity Assay

Cytotoxicity was evaluated using the Promega Viral ToxGlo Assay (Promega, Madison, WI, USA) following the manufacturer’s protocol. Vero and Huh-7 cells were incubated with RBV or IFN for three days. Plates were read using Promega Glomax 96 microplate luminometer (Promega) and the concentration-effect relationship was determined by fitting an inhibitory sigmoid-E_max_ model to log transformed relative light unit (RLU) measurements using GraphPad Prism software (GraphPad Software, San Diego, CA, USA).

### 2.3. Antiviral Evaluations

The antiviral activity of RBV and/or IFN was evaluated as single agent and combination therapy against DENV2 on Vero and Huh-7 cells, as previously described [20]. Briefly, confluent cell monolayers in 6-well plates were infected with DENV2 at a multiplicity of infection (MOI) of 0.001 PFU/cell for Vero cells and 0.01 PFU/cell for Huh-7 cells and incubated for 1 h at 37 °C, 5% CO_2_. Due to differences in viral replication kinetics between cell lines, the MOI for each cell line was selected to yield similar viral burden profiles over time. Monotherapy supernatant samples were collected daily for four days in Vero cells and three days in Huh-7 cells. For the combination assays, supernatants were collected at day 3 post-treatment for Vero cells and day 2 post-treatment for Huh-7 cells, corresponding to the time point in which peak of viral burden was achieved in the control. Samples were clarified and frozen at −80 °C until the end of study. Infectious viral burden was determined for all samples simultaneously by plaque assay on Vero cells. All assays were performed two times in triplicate.

### 2.4. DENV Plaque Assay

Infectious viral burden of supernatant samples was quantified by plaque assay on Vero cells in six-well plates. Supernatant samples were serially diluted 10-fold in MEM containing 2% FBS and 100 µL of each dilution was inoculated onto confluent Vero cell monolayers. Cells were incubated for 1 h at 37 °C, 5% CO_2_. A primary overlay with a final concentration of 0.6% agar, MEM, and 5% FBS was added to cell monolayers and incubated for five days at 37 °C, 5% CO_2_. After five days, a secondary agar overlay was added containing a final concentration of 1% agar containing MEM, 1% FBS, 200 μg/mL Diethylaminoethyl-dextran, and 0.008% Neutral red. Plates were incubated overnight and plaques were counted the next day. Viral burden is reported as log_10_ plaque forming units per ml (PFU/mL).

### 2.5. Statistical Analysis

For monotherapy, *EC*_50_ values were determined over the entire time course of the assay by calculating the area under the viral burden-time curve (AUC_viral_burden_) for all monotherapy arms. An inhibitory sigmoid-E_max_ model was fit to the AUC_viral_burden_ values. Analyses were conducted using GraphPad Prism software.

The Greco Universal Response Surface Approach (URSA) model [23] was used to determine drug-drug interactions for anti-DENV effect between IFN and RBV as a combination therapy, as previously described [24]. Briefly, the Greco URSA model is shown below:

1=D1EC50 D1(EEcon−E)1m1+D2EC50 D2(EEcon−E)1m2+αD1D2EC50 D1 EC50 D2 (EEcon−E)12m1+12m2

*E* represents the measured antiviral effect, *D*_1_ corresponds to RBV concentration, *D*_2_ is IFN concentration, *EC*_50_ *_D_*_1_ is the concentration of RBV resulting in half maximal antiviral effect, and *EC*_50_ *_D_*_2_ is the concentration of IFN resulting in half maximal antiviral effect. *m*_1_ refers to the Hill’s constant for RBV and m_2_ signifies the Hill’s constant for IFN. *E_con_* represents the viral burden in the absence of drug (the control response) and *α* is the drug-drug interaction parameter. Data were analyzed using the ID module of ADAPT software (version 5, Biomedical Simulations Resrouce, Los Angeles, CA, USA).

### 2.6. Delayed Treatment Studies in the HFIM System

The influence of RBV and IFN therapy initiation on antiviral effect was assessed in the HFIM system. The HFIM system is described in detail elsewhere [24,25,26,27,28]. Briefly, 10^8^ Huh-7 cells were mixed with 10^5^ PFU of DENV2 (MOI = 0.001 PFU/cell) in cell culture media and inoculated into a cellulosic hollow fiber cartridge (FiberCell Systems, Frederick, MD, USA). Six cartridges were employed for this study. One cartridge did not receive drug treatment and served as a no-treatment control. IFN plus RBV therapy was added to the five remaining cartridges, but treatment was initiated at different time-points post-cartridge inoculation (0, 2, 6, 12 and 24 h). Human pharmacokinetic (PK) profiles associated with the HCV clinical regimen of IFN at 36 million IU twice daily (BID) in combination with 600 mg BID of RBV was used to calculate the average free-drug concentrations (C_avg_) in human plasma every 24 h over three days of treatment (Table 1) [29,30]. Cells were exposed to IFN plus RBV combination therapy for three days. Supernatant samples were collected daily, clarified by high-speed centrifugation, and stored at −80 °C. Viral burden was quantified by plaque assay on Vero cells.

### 2.7. Antiviral Activity of Clinical Exposures of IFN and RBV after 24 h Post-Infection in the HFIM System

Four cellulosic hollow fiber cartridges were inoculated with 10^8^ Huh-7 cells/cartridge and mixed with DENV2 at a MOI of 0.001 PFU/cell. After 24 h post-infection (p.i.), exposures associated with the clinical dose of RBV (600 mg BID) and IFN (36 million IU BID) (Table 1) [29,30] were administered into four cartridges for three days as single agents and in combination. One cartridge served as a no-treatment control. Samples were collected daily clarified by high speed centrifugation and stored at −80 °C until viral burden was quantified by plaque assay on Vero cells.

## 3. Results

### 3.1. Monotherapy Antiviral Activity and Cytotoxicity

The antiviral activity of RBV and IFN was first evaluated as single agents against DENV in Vero (monkey kidney cell) and Huh-7 (human liver cell) cells. Infected cells were exposed to compounds for 4 days in Vero cells or three days in Huh-7 cells, as we chose the day after peak viral burden as the endpoint of the assay. *EC*_50_ values were calculated over the entire duration of the experiment via a Hill model. RBV exhibited an *EC*_50_ value of 106.6 mg/L and a 50% cytotoxicity concentration (*CC*_50_) value of 137.4 mg/L in Vero cells. These values are substantially higher than those reported for Huh-7 cells, which yielded an *EC*_50_ value of 10.02 mg/L and a *CC*_50_ value of 18.0 mg/L (Table 2). For both cell lines, RBV antiviral activity was modest with *EC*_50_ values similar to the reported *CC*_50_ values. These findings suggest that RBV antiviral activity is mainly due to cytotoxicity. On the other hand, IFN showed a greater degree of viral suppression compared to RBV, with *EC*_50_ values far below the *CC*_50_ values. IFN yielded an *EC*_50_ value of 1381 IU/mL in Vero cells and 58.34 IU/mL in Huh-7 cells, with *CC*_50_ values greater than 10,000 IU/mL for both cell lines (Table 2).

### 3.2. Combination Therapy against DENV in a Plate Assay

**Figure 1:**
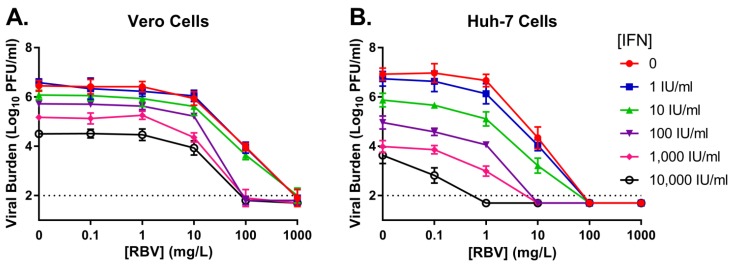
Antiviral activity of ribavirin (RBV) and interferon-α (IFN) as a combination therapy against DENV2. (**A**) Vero cells were infected at a multiplicity of infection of 0.001 PFU/cell and exposed to RBV and/or IFN. Viral supernatants were collected on day 3; (**B**) Huh-7 cells were infected at a multiplicity of infection of 0.01 PFU/cell and exposed to RBV and/or IFN. Supernatant samples were harvested after two days post-infection. Viral burden was quantified by plaque assay in Vero cells and reported as log_10_ PFU/mL. Data points correspond to the geometric mean of three independent samples and error bars represent one standard deviation. Horizontal dashed lines represent the assay limit of detection.

IFN and RBV were evaluated as combination therapy against DENV to examine whether administration of two drugs together would enhance antiviral activity and/or make either agent more effective at lower concentrations. Peak viral titers were similar between cell lines in the control regimens, yielding a viral burden of 6.5 log_10_ PFU/mL for Vero cells and 6.9 log_10_ PFU/mL for Huh-7 cells (Figure 1). The addition of RBV to IFN did enhance antiviral activity relative to monotherapy arms in both Vero and Huh-7 cell lines; however, increased effectiveness was most obvious when very high concentrations of RBV were present. Huh-7 cells were more susceptible to the IFN and RBV combination regimen, as similar exposures of IFN plus RBV consistently yielded greater extents of viral suppression compared to Vero cells (Figure 1). The cytotoxicity profile of the combination regimen was identical to that reported for the RBV and IFN monotherapy regimens. 

To identify the drug-drug interactions (i.e., synergy, additivity, or antagonism) between RBV and IFN on Vero and Huh-7 cells, the Greco URSA model was employed and fit to the data illustrated in Figure 1. The model fits were precise and unbiased, resulting in *r*^2^ values of 0.928 for Vero cells and 0.924 for Huh-7 cells (Table 3). The *EC*_50_ values were 69.41 mg/L for RBV and >10,000 IU/mL for IFN in Vero cells on day 3 post-infection (Table 3). *EC*_50_ estimates were markedly lower in Huh-7 cells which resulted in values of 6.06 mg/L for RBV and 110.2 IU/mL for IFN on day 2 post-infection (Table 3). The drug interaction parameter, alpha (α), was positive for both cell lines with final estimates of 22.86 (95% CI, −11.30–57.03) for Vero cells and 34.87 (95% CI, −30.54–100.3) for Huh-7 cells. Since the 95% confidence intervals surrounding both α estimates overlap zero, RBV in combination with IFN results in additivity for the suppression of infectious DENV when Vero cells and Huh-7 cells are employed as the host cell line.

### 3.3. Delayed Treatment Studies in the HFIM System

We evaluated the antiviral activity of RBV and IFN against DENV as combination therapy at clinically relevant exposures using the HFIM system. As second objective, we also examined the window of treatment initiation for the combination regimen and its impact on antiviral effectiveness against DENV. Evaluations were only conducted in Huh-7 cells due to the observed increase in sensitivity to drug treatment in this line relative to Vero cells and that these cells are of human origin. Huh-7 cells were mixed with DENV and RBV plus IFN was added at 0 h (immediately), 2 h, 6 h, 12 h, and 24 h post-inoculation (p.i.) in the HFIM system.

**Figure 2:**
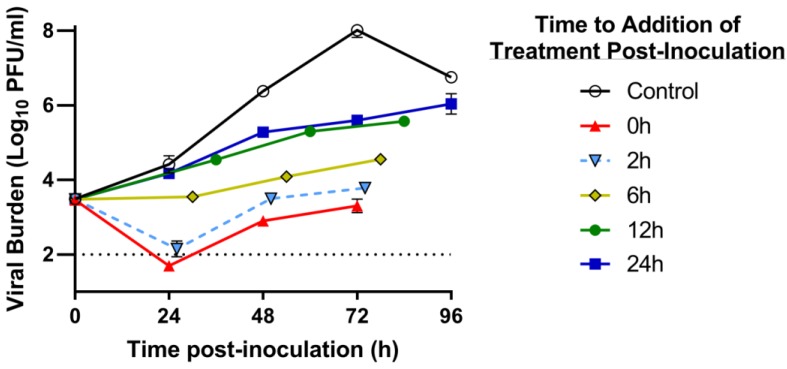
The influence of time to initiation of treatment on the antiviral effect of ribavirin (RBV) plus Interferon-α (IFN) when administered at exposures associated with the clinical regimens against DENV2 in the Hollow fiber infection model (HFIM) system. Huh-7 cells were mixed with DENV at a multiplicity of infection of 0.001 PFU/cell and inoculated in the HFIM. Drug exposure was the same between all treatment arms, with the exception of the control (untreated) arm. The combination regimen was administered into HF cartridges at different times post-inoculation (0 h, 2 h, 6 h, 12 h, and 24 h). Supernatant samples were collected from the system for three days following drug administration and viral burden was quantified by plaque assay on Vero cells. Viral burden is reported as log_10_ PFU/mL. Each data point represents the geometric mean of two independent samples and error bars correspond to one standard deviation. The assay limit of detection is represented by the dashed horizontal line.

In the absence of drug, DENV exhibited robust replication kinetics in the HFIM system, achieving peak viral titers of 8.0 log_10_ PFU/mL at 72 h p.i. (Figure 2). DENV inhibition by clinically-relevant exposures of RBV plus IFN was dependent on the time of treatment initiation, as the degree of suppression was markedly increased with earlier administration of the combination. Administration of RBV and IFN at 0 h or 2 h p.i. into the HFIM system reduced peak viral burden by 4.7 log_10_ PFU/mL and 4.2 log_10_ PFU/mL, respectively, relative to the control (Figure 2). Conversely, viral titers were inhibited by 2.4 log_10_ PFU/mL and 2 log_10_ PFU/mL when the combination was administered 12 h and 24 h p.i., respectively (Figure 2). These results show that RBV plus IFN therapy at clinically achievable concentrations has the potential to reduce infectious DENV by 99%, even when drug administration was delayed 24 h.

### 3.4. Antiviral Evaluations of Clinical Exposures of IFN and/or RBV after 24 h p.i. in the HFIM System

**Figure 3:**
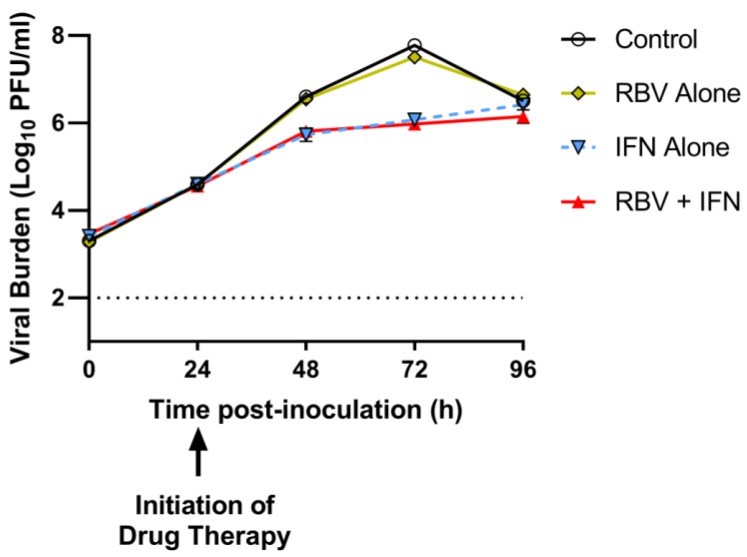
Antiviral activity of clinical exposures of interferon-α (IFN) and/or ribavirin (RBV) after 24 h post-infection (p.i.) in the Hollow fiber infection model (HFIM) system. Huh-7 cells were mixed with DENV2 at a multiplicity of infection of 0.001 PFU/cell and inoculated into the HFIM system. After 24 h post-inoculation, cells were exposed to RBV and/or IFN at levels corresponding to the clinical regimen. Supernatants were collected from the system for three days after the start of drug administration. Viral burden was quantified by plaque assay in Vero cells and is described as log_10_ PFU/mL. Each data point represents the geometric mean of two independent samples and error bars (smaller than symbols and, thus, not visible) correspond to one standard deviation. The dashed horizontal line represents the assay limit of detection.

RBV and IFN were evaluated at clinically-relevant exposures as mono- and combination therapy in the HFIM system to identify the antiviral activity of each agent against DENV when treatment was initiated 24 h p.i. RBV alone at exposures associated with the clinical dose of 600 mg BID (Table 1) was not effective at suppressing DENV, as viral titers were nearly identical to those reported for the control over the entire duration of the experiment (Figure 3). Conversely, IFN monotherapy inhibited viral burden by 1.7 log_10_ PFU/mL, a value that is strikingly similar to the extent of inhibition provided by the combination regimen (1.8 log_10_ PFU/mL) at 72 h p.i. (Figure 3). These findings indicate that the antiviral activity of RBV and IFN as combination therapy is mostly attributed to the effect of IFN against DENV.

## 4. Discussion

DENV is a major public health challenge with approximately 2.5 billion people at risk of infection every year [31]. The development of safe and effective vaccines against DENV is complicated due to the existence of four distinct DENV serotypes, as the vaccine must protect against each serotype equally to be successful. Failure to mount a robust immune response to each serotype may exacerbate disease upon infection due to ADE [7,32]. The challenge of vaccine development has been recently highlighted by the significant safety concerns associated with the use of the Sanofi Dengvaxia^®^ vaccine in DENV-naïve individuals [12]. These concerns have caused some countries to restrict the use of the vaccine or have recalled the vaccine altogether. Moreover, the discovery and potential spread of a new fifth DENV serotype will likely add to the hurdles facing vaccine development and further delay approval for human use [4]. These problems associated with vaccination illustrate the crucial need for additional medical countermeasures against DENV, including effective antiviral regimens. Here, we evaluated the clinical potential of RBV and IFN as combination therapy, an approved regimen for the treatment of HCV-infected patients, against DENV.

RBV has been shown to have limited effectiveness against DENV in preclinical studies when a variety of different host cells were utilized [33,34]. Our findings agree with these earlier reports, as RBV demonstrated minimal activity in both Vero and Huh-7 cells at non-toxic concentrations. Although substantial anti-DENV effect was observed at RBV concentrations ranging from 100–1000 mg/L for Vero cells and 10–1000 mg/L for Huh-7 cells, these concentrations are similar to or exceed the corresponding *CC*_50_ values and suggest that antiviral activity is directly related to host cell toxicity and not direct antiviral effect. Moreover, Preston et al. showed that the maximum concentration (C_max_) of RBV in healthy volunteers after intravenous administration is 4.187 mg/L with a steady-state C_ave_ of approximately 1 mg/L [30]. Both concentrations are markedly lower than the *EC*_50_ values reported against DENV. Together these results imply that RBV as monotherapy holds no clinical promise against DENV due to the fact that it is only effective at supratherapeutic concentrations that are not achievable in humans due to toxicity.

In contrast to RBV, others have shown that IFN monotherapy exhibits anti-DENV activity in vitro [18,35]. Our findings support these studies, as IFN alone inhibited 99% of DENV burden at 100 IU/mL on Vero cells and 10 IU/mL on Huh-7 cells on the day of peak viral burden in the control (Figure 1). Moreover, the *EC*_50_ value for IFN against DENV was 58.34 IU/mL on Huh-7 cells over the course of three days of therapy (Table 2). This level of IFN is physiologically achievable, as the clinical regimen of 36 million IU (MIU) BID is associated with a systemic steady-state C_max_ of 315 IU/mL and a C_ave_ of 263.3 IU/mL [29]. Thus, our results suggest that clinically-relevant concentrations of IFN are effective at suppressing infectious virus on host cells derived from human tissue, demonstrating the clinical potential of this agent as a treatment strategy for DENV.

Despite the promising antiviral activity of IFN against DENV on Huh-7 cells, similar findings were not observed when antiviral evaluations were conducted on Vero cells. Although IFN inhibited infectious DENV by 2 log_10_ PFU/mL on day 3 (day of peak viral titer in the control; Figure 1), viral suppression was not sustained and resulted in an overall *EC*_50_ value of 1381 IU/mL (Table 2). This value is approximately five times higher than physiologically-achievable concentrations [29]. The discordance in results between Huh-7 and Vero cells is likely explained by the fact that Vero cells are deficient for interferon-stimulating genes, rendering these cells incapable of synthesizing endogenous IFN [36,37,38]. Vero cells are able to respond to exogenous IFN because IFN receptors are present on the cell surface. Huh-7 cells, on the other hand, have the capacity to respond to exogenous IFN as well as produce endogenous IFN. Thus, we hypothesize that the decreased antiviral effect of IFN on Vero cells is directly related to IFN deficiency that is associated with this cell line. These findings illustrate the importance of proper host cell selection for preclinical drug evaluations.

The addition of RBV to IFN regimens has been shown to significantly improve treatment outcomes in patients infected with HCV [22,39], indicating that RBV and IFN have a positive interaction for antiviral effect. We explored whether the combination of RBV plus IFN would enhance the anti-DENV activity observed for IFN. Our results showed that these two agents are additive for DENV suppression on Vero and Huh-7 cell lines, as determined by the Greco URSA model. However, it is important to note that this designation is somewhat misleading. Our antiviral evaluations on Vero cells showed that IFN anti-DENV activity was only enhanced when RBV concentrations in excess of 10 mg/L were present (Figure 1). These RBV concentrations are supratherapeutic and are not clinically appropriate for DENV treatment. Conversely, the extent of inhibition by IFN on Huh-7 cells was greater when combined with at least 1 mg/L of RBV, a clinically-relevant concentration. The physiological achievable combination of 1 mg/L RBV plus 100 IU/mL IFN reduced DENV burden by 3 log_10_ PFU/mL, yielding an additional 1 log_10_ PFU/mL and 2.7 log_10_ PFU/mL decrease in viral titer relative to IFN and RBV monotherapy, respectively. These findings demonstrate that RBV has potential to enhance the antiviral activity of IFN against DENV when Huh-7 cells are employed as the host cell line and that this combination regimen may have clinical benefit. These results are encouraging as Huh-7 cells are likely a more relevant tissue culture model for DENV infection for three reasons: (1) they are of human origin; (2) liver involvement has been frequently described in human DENV infections, suggesting that liver tissue may be infected with virus [40,41]; (3) they are proficient in responding to and synthesizing endogenous IFN. Thus, we further investigated RBV plus IFN as a potential treatment strategy for DENV in the HFIM system.

The antiviral activity of free-drug exposures associated with the standard clinical regimen of RBV (oral delivery 600 mg BID) and IFN (injection of 36 MIU BID) administered as combination therapy over three days against DENV in Huh-7 cells was evaluated using the HFIM system. In our initial studies, cells were mixed with virus and exposed to drug treatment simultaneously in the HF cartridge. This experimental design resulted in extensive viral suppression (nearly 5 log_10_ PFU/mL; Figure 2), which was improbable based on our plate assay results. We hypothesized this degree of suppression was due to the mechanism of action of IFN which creates an antiviral state in uninfected cells, making them refractory to infection. Thus, we altered our experimental protocol to delay initiation of treatment to allow for Huh-7 cells to become infected with DENV prior to drug administration. We found that clinically-relevant exposures of RBV plus IFN were able to suppress DENV replication by 99% even when treatment was started 24 h p.i. However, a more in-depth analysis revealed that the antiviral activity associated with the combination regimen was solely due to the action of IFN. The addition of RBV provided no clinical benefit when administered at C_ave_ concentrations corresponding to the clinical dose (Table 1) and only increases the risk of toxicity. These findings suggest that IFN monotherapy is the most promising antiviral regimen of those examined in this work for the treatment of DENV.

There are several limitations to our study. First, selecting the appropriate host cell line for antiviral evaluations is crucial, since different cell lines can yield different conclusions regarding effectiveness (as demonstrated here). Although clinical data indicate that the liver is affected during human DENV infection, the true clinical relevance of this tissue is unknown. We are currently evaluating DENV replication kinetics in several other human cell lines derived from other, perhaps more relevant, tissues, including blood, skin, and brain tissue. We will repeat antiviral evaluations in a line that is permissive to DENV infection and yields robust replication kinetics. A second limitation is that only one serotype of DENV was assessed in this work. Future studies are underway to evaluate this combination regimen against other DENV serotypes. Thirdly, all of our drug assays were conducted in the presence of static drug concentrations. It is possible that simulating dynamic human pharmacokinetic profiles of RBV, in which C_max_ values of at least 4 mg/L will be achieved, may result in additional anti-DENV activity. Moreover, evaluating higher dose RBV regimens which have been used against other viral infections, including Lassa virus, could also lead to greater viral inhibition. These studies are currently ongoing in our laboratory. Finally, it is worth noting that our experimental system does not account for the immune system. The inclusion of a functioning immune component in addition to IFN therapy would likely increase viral suppression, further reducing viral burden.

## 5. Conclusions

Here, we have demonstrated that clinical exposures of IFN have potential to be used as a treatment against DENV infections, as it may be possible to achieve sufficient levels of viral inhibition at physiologic drug exposures. Our interesting findings indicate that further investigations using dynamic concentrations of RBV in combination with IFN should be conducted to better evaluate the potential of this combination therapy against DENV.

## Figures and Tables

**Table 1:** Average concentrations (C_avg_) calculated based on the human pharmacokinetics (PK) profiles associated with the HCV clinical regimens of 36 million IU twice daily (BID) of interferon-α (IFN) and 600 mg BID of ribavirin (RBV).

Time (day)	IFN C_avg_ (IU/mL) ^a^	RBV C_avg_ (mg/L) ^b^
1	182.10	0.394
2	250.78	0.616
3	259.88	0.76

^a^ Concentrations based on [29]; ^b^ Concentrations based on [30].

**Table 2:** Cytotoxicity and antiviral activity of Ribavirin (RBV) and Interferon-α (IFN) as single agents on Vero and Huh-7 cells.

		Vero ^a^		Huh-7 ^a^		
	Parameter	RBV	IFN	RBV	IFN	Units
**Antiviral Effect**	*r* ^2 b^	0.999	0.986	0.915	0.973	--
	*E_con_* ^c^	6.46	6.61	6.82	7.04	Log_10_ PFU/mL
	*EC*_50_^d^	106.6	1381	10.02	58.34	mg/L or IU/mL
	*H* ^e^	0.934	0.356	14.44	0.315	--
**Cytotoxicity**	*r* ^2 b^	0.995	--^f^	0.958	--^f^	--
	*E_con_* ^c^	7.53	7.54	7.47	7.40	Log_10_ RLU
	*CC*_50_^d^	137.4	>10,000	18.0	>10,000	mg/L or IU/mL
	*H* ^e^	1.87	--^f^	1.76	--^f^	

^a^ The *CC*_50_ and *EC*_50_ values were calculated using GraphPad Prism software. The data are representative of three independent experiments; ^b^ *r*^2^ is coefficient of determination that quantifies goodness of fit; ^c^ *E_con_* is a measure of effect in the absence of drug (control); ^d^ *EC*_50_ and *CC*_50_ values are concentrations of RBV or IFN resulting in 50% of maximal effect. RBV *EC*_50_/*CC*_50_ values are reported in mg/L and IFN *EC*_50_/*CC*_50_ values are reported in IU/mL; ^e^ *H* is the Hill’s constant; ^f^ Log_10_ RLU output was not changed from baseline (no-treatment) in the presence of IFN, even at the highest concentrations evaluated. Thus, an inhibitory sigmoid Emax model could not be fit to the data and *CC*_50_ values were estimated at >10,000 IU/mL. --: Parameter estimate does not have units associated with the value.

**Table 3:** Mean estimate parameter values from ribavirin (RBV) and interferon-α (IFN) combination therapy.

Parameter	Final Estimate		Units
	Vero	Huh-7	
***r*^2 a^**	0.928	0.924	--
***E_con_*^b^**	6.072	6.73	Log_10_ PFU/mL
***EC*_50 *RBV*_^c^**	69.41	6.06	mg/L
***m*_1_^d^**	1	1	--
***EC*_50 *IFN*_^e^**	>10,000	110.2	IU/mL
***m*_2_^f^**	1	1	--
***α*^g^**	22.86 [−11.3–57.03] ^h^	34.87 [−30.54–100.3] ^h^	--

^a^*r*^2^ is coefficient of determination that quantifies goodness of fit; ^b^ *E_con_* is a measure of effect (or response) of the control; ^c^ *EC*_50 *RBV*_ is the concentration of drug resulting in 50% of effect of ribavirin (RBV); ^d^ *m*_1_ is the Hill’s constant for RBV; ^e^ *EC*_50 *IFN*_ is the concentration of drug resulting in 50% of effect of interferon-α (IFN); ^f^ *m*_2_ is the Hill’s constant for IFN; ^g^ *α* is the interaction parameter; ^h^ 95% confidence interval around the α parameter; --: Parameter estimate does not have units associated with the value.
